# Association of PAX2 and Other Gene Mutations with the Clinical Manifestations of Renal Coloboma Syndrome

**DOI:** 10.1371/journal.pone.0142843

**Published:** 2015-11-16

**Authors:** Toshiya Okumura, Kengo Furuichi, Tomomi Higashide, Mayumi Sakurai, Shin-ichi Hashimoto, Yasuyuki Shinozaki, Akinori Hara, Yasunori Iwata, Norihiko Sakai, Kazuhisa Sugiyama, Shuichi Kaneko, Takashi Wada

**Affiliations:** 1 Department of Disease Control, Institute of Medical, Pharmaceutical and Health Sciences, Faculty of Medicine, Kanazawa University, Kanazawa, Japan; 2 Division of Blood Purification, Kanazawa University Hospital, Kanazawa, Japan; 3 Departments of Ophthalmology and Visual Science, Institute of Medical, Pharmaceutical and Health Sciences, Faculty of Medicine, Kanazawa University, Kanazawa, Japan; 4 Division of Nephrology, Department of Laboratory Medicine, Institute of Medical, Pharmaceutical and Health Sciences, Faculty of Medicine, Kanazawa University, Kanazawa, Japan; University of Iowa, UNITED STATES

## Abstract

**Background:**

Renal coloboma syndrome (RCS) is characterized by renal anomalies and optic nerve colobomas. *PAX2* mutations contribute to RCS. However, approximately half of the patients with RCS have no mutation in *PAX2 gene*.

**Methods:**

To investigate the incidence and effects of mutations of *PAX2* and 25 candidate genes, patient genes were screened using next-generation sequence analysis, and candidate mutations were confirmed using Sanger sequencing. The correlation between mutations and clinical manifestation was evaluated.

**Result:**

Thirty patients, including 26 patients (two families of five and two, 19 sporadic cases) with RCS, and 4 optic nerve coloboma only control cases were evaluated in the present study. Six *PAX2* mutations in 21 probands [28%; two in family cohorts (n = 5 and n = 2) and in 4 out of 19 patients with sporadic disease] including four novel mutations were confirmed using Sanger sequencing. Moreover, four other sequence variants (CHD7, SALL4, KIF26B, and SIX4) were also confirmed, including a potentially pathogenic novel KIF26B mutation. Kidney function and proteinuria were more severe in patients with *PAX2* mutations than in those without the mutation. Moreover, the coloboma score was significantly higher in patients with *PAX2* gene mutations. Three out of five patients with *PAX2* mutations had focal segmental glomerulosclerosis (FSGS) diagnosed from kidney biopsies.

**Conclusion:**

The results of this study identify several new mutations of PAX2, and sequence variants in four additional genes, including a novel potentially pathogenic mutation in KIF26B, which may play a role in the pathogenesis of RCS.

## Introduction

Renal coloboma syndrome (RCS; papillorenal syndrome) is characterized by kidney hypoplasia or dysplasia and abnormality of the optic nerve. Abnormal kidney structure and function vary among patients. For example, some abnormalities are clinically silent but others include kidney insufficiency and end-stage renal disease. Further, patients with RCS exhibit ocular abnormalities. Developmental abnormalities of the optic nerve (ranging from mild optic disc dysplasia to optic nerve aplasia), retina, choroid, and iris are included in coloboma [1]. Abnormal eye structure and function, such as serious retinal detachment and thin hypoplastic peripheral retinas, also vary among patients [2]. Although the diagnosis of RCS is based on the morphological examination of the kidney, ophthalmologic findings, and family history, the variations of clinical manifestations make timely diagnosis difficult.

*PAX2* mutations represent one of the main genetic abnormalities of RCS [3, 4]. *PAX2* encodes a transcription factor that mediates development and plays key roles in different stages of the development of the kidneys, eyes, ears, and genital tract [5]. Studies of animals with *PAX2* mutations show congenital loss of nephron number [2, 4–6].Approximately 170 cases with *PAX2* gene abnormalities have been reported in cases with RCS worldwide [7]. However, around 50% cases of RCS have no abnormality in *PAX2 gene*[8].

*PAX2* is a key transcriptional factor that functions in the regulation of a diverse range of genes involved in kidney and eye development. A previous study using RNA-seq analysis following *PAX2* siRNA knockdown identified 3135 transcripts differentially regulated by *PAX2* [9]. *PAX2*-mediated gene expression is reportedly regulated by many transcriptional and intra-cellular signaling factors including Wnt ligands, bone morphogenic proteins, fibroblast growth factor, sonic hedgehog, RET/glial cell-derived neurotrophic factor, notch signaling pathways [9], *PAX2*-binding proteins such as Hox11 and Eya1 [10], and epigenetic modulation of target genes regulated by PTIP and the mixed-lineage leukemia (MLL) complex [11, 12]. Moreover, kidney and eye development is regulated by a complex network of genes, including *GDF11*, *GDNF*, *FOXC1*, *SIX1*, *SALL1*, *PAX8*, and *WT1*) [13]. Among them, we selected 25 candidate genes. Therefore, we speculated mutations in our candidate genes, in addition to *PAX2* mutations, contribute to RCS pathogenesis.

In the present study, we hypothesized that *PAX2* and 25 candidate genes contribute to the pathogenesis of RCS. Here we report the evaluation of 26 patients, including two family cohorts (n = 5 and n = 2) and 19 patients with sporadic disease for mutations in *PAX2* and 25 candidate genes. We have detected four novel PAX2 mutations in 11 patients, and a potentially pathogenic KIF26B mutation in one patient. Our results suggest that these mutations may contribute to the pathogenesis of RCS.

## Methods

### Patients

Thirty patients, including two family cohorts (n = 5 and n = 2), 19 patients with sporadic RCS, and 4 coloboma only control cases were evaluated in the present study. Although the diagnosis of RCS is established according to clinical studies of the kidneys and eyes, there are no formal diagnostic criteria for RCS [14]. In the present study, we enrolled patients with kidney dysfunction, renal morphological abnormalities, or both. Optic nerve coloboma (dysplasia of the optic nerve) was defined as a clearly demarcated bowl-shaped excavation of the optic disc, which is typically decentered and deep. Slit-lamp examination, intraocular pressure measurement, fundus examination, and photography as well as measurement of visual acuity were performed. In addition to these clinical findings on the kidney and eyes, similar other syndromes were excluded by clinical evaluation. Patients with CHARGE syndrome (coloboma, heart malformations, atresia choanae, retardation of growth and development, genital anomalies, and ear and hearing abnormalities) were excluded according to the absence of the typical CHARGE syndrome findings of craniofacial abnormalities or cognitive difficulties. Patients with COACH syndrome (cerebral vermis hypoplasia, oligophrenia, ataxia, optic nerve coloboma, hepatic fibrosis) and Joubert syndrome were excluded according to the absence of the typical COACH and Joubert syndrome findings of developmental disability, cerebellar hypoplasia, cerebellar dysfunction, and hepatic dysfunction. We analyzed the sequences of PAX2 and 25 other genes in 26 patients clinically diagnosed with RCS, and 4 optic nerve coloboma only patients as disease-negative controls.

### Ethics statement

The Ethics Committee of the Kanazawa University Graduate School of Medicine approved the study (Approval No. 169). All analyses in this study conformed to the ethical guidelines of the 1975 Declaration of Helsinki in its respective latest version. All individuals were >18 years of age. Written informed consent was obtained from all participants.

### Nucleotide sequence analysis

Screening of DNA sequences was conducted using an Ion Torrent PGM sequencer equipped with the Ion 318 chip. We obtained sequences covering >90% of all genes, and the mean coverage was 97.7% (Table 1). Genomic positions of the mutations were determined using human genome build 19 and the University of California, Santa Cruz genome annotation database http://genome.ucsc.edu. The CLC Genomics Workbench was used to further process the sequence alignments and genotyping data. Variants were filtered using data from dbSNP 142 and the 1000 Genomes Project.

**Table 1:** DNA screening by next-generation sequencing.

	Name	Target (bp)	Missed (bp)	Covered (%)
**1**	**WNT4**	**1111**	**88**	**92.1**
**2**	**SIX2**	**898**	**13**	**98.6**
**3**	**SIX1**	**877**	**0**	**100.0**
**4**	**SALL4**	**3206**	**0**	**100.0**
**5**	**SALL1**	**7266**	**87**	**98.8**
**6**	**NOTCH2**	**7987**	**139**	**98.3**
**7**	**FOXC1**	**1673**	**163**	**90.3**
**8**	**PAX8**	**1595**	**0**	**100.0**
**9**	**HNF1B**	**1971**	**0**	**100.0**
**10**	**EYA1**	**2125**	**0**	**100.0**
**11**	**HOX11**	**1041**	**14**	**98.7**
**12**	**RET**	**3756**	**8**	**99.8**
**13**	**PAX2**	**1602**	**0**	**100.0**
**14**	**KIF26B**	**6492**	**238**	**96.3**
**15**	**HESR1**	**1077**	**0**	**100.0**
**16**	**BMP7**	**1373**	**0**	**100.0**
**17**	**BMP4**	**1249**	**0**	**100.0**
**18**	**WT1**	**1780**	**76**	**95.7**
**19**	**WNT9A**	**1142**	**106**	**90.7**
**20**	**CHD7**	**9401**	**28**	**99.7**
**21**	**WNT9B**	**1118**	**81**	**92.8**
**22**	**LHX1**	**1276**	**84**	**93.4**
**23**	**GDNF**	**1076**	**6**	**99.4**
**24**	**NOTCH1**	**8042**	**106**	**98.7**
**25**	**JAG1**	**3943**	**10**	**99.8**
**26**	**SIX4**	**2379**	**65**	**97.3**
	**Mean**	**2902.1**	**50.5**	**97.7**

Gene mutations were screened using next-generation sequence analysis.

All genes were covered more than 90%, and the mean coverage was 97.7%.

*PAX2* gene mutations were evaluated according to our published method[15]. Twelve primer sets for each *PAX2* exon were synthesized according to sequence information with some modifications [16]. Amplified DNA fragments were purified and sequenced using a dye terminator cycle sequencing kit (DYEnamic ET Terminator; GE Healthcare Bio-Sciences Corp., Piscataway, NJ) and an automated DNA sequencer (ABI PRISM 310 DNA Sequencer; Applied Biosystems, Foster City, CA). Candidate mutations of the 25 other genes were determined using Sanger sequencing with same primers used for the Ion Torrent PGM sequencer at TaKaRa Dragon Genomics Center. Mutation Taster (http://www.mutationtaster.org) was used to predict potential contributions of individual mutations to disease. Moreover, allele frequencies were evaluated using the ExAc database (http://exac.broadinstitute.org).

### Kidney biopsy specimens

Kidney biopsies were obtained from eight patients. For pathological diagnosis, a portion of each kidney specimen was fixed with 10% phosphate-buffered formalin (pH 7.4), embedded in paraffin, cut into 4-μm thick sections, and stained with hematoxylin and eosin, periodic acid-Schiff’s reagent, periodic acid silver methenamine, and Mallory-Azan solutions.

### Scoring of optic disc coloboma

An experienced ophthalmologist (T. H.) reviewed photographs of the fundus of both eyes of each patient to determine the presence of an optic disc coloboma and other abnormalities of the fundus. The optic disc coloboma was scored according to a 5-point scale of 0 (normal) to 4 (severe) [8] as follows: 0 = normal; 1 = optic disc dysplasia, with an unusual pattern of retinal vessels and cilioretinal arteries; 2 = optic disc pit associated with vascular abnormalities and a cilioretinal artery; 3 = large coloboma involving the entire surface of the optic disc; 4 = large coloboma of the optic disc and adjacent retina or morning-glory anomaly (with radial emergence of the retinal vessels).

### Statistical analysis

SPSS 19.0 and the StatView program were used for statistical analysis. Data are expressed as the mean ± standard error (mean ± SEM). Statistical analyses were performed using the Mann–Whitney U test; p < 0.05 indicated a statistically significant difference.

## Results

### Detection of mutations in patients with RCS

Screening the sequences of *PAX2* and 25 other genes identified 760 SNPs and 9 small indels. Among them, 162 SNPs and 9 small indels were missense mutations. Moreover, 116 SNPs were deleted by filtering using the dbSNP137 and the 1000 Genomes Project database. Of these, 46 non-synonymous SNPs and 9 indels shared by the affected individuals were retained in the analysis (Table 1).

**Fig 1:**
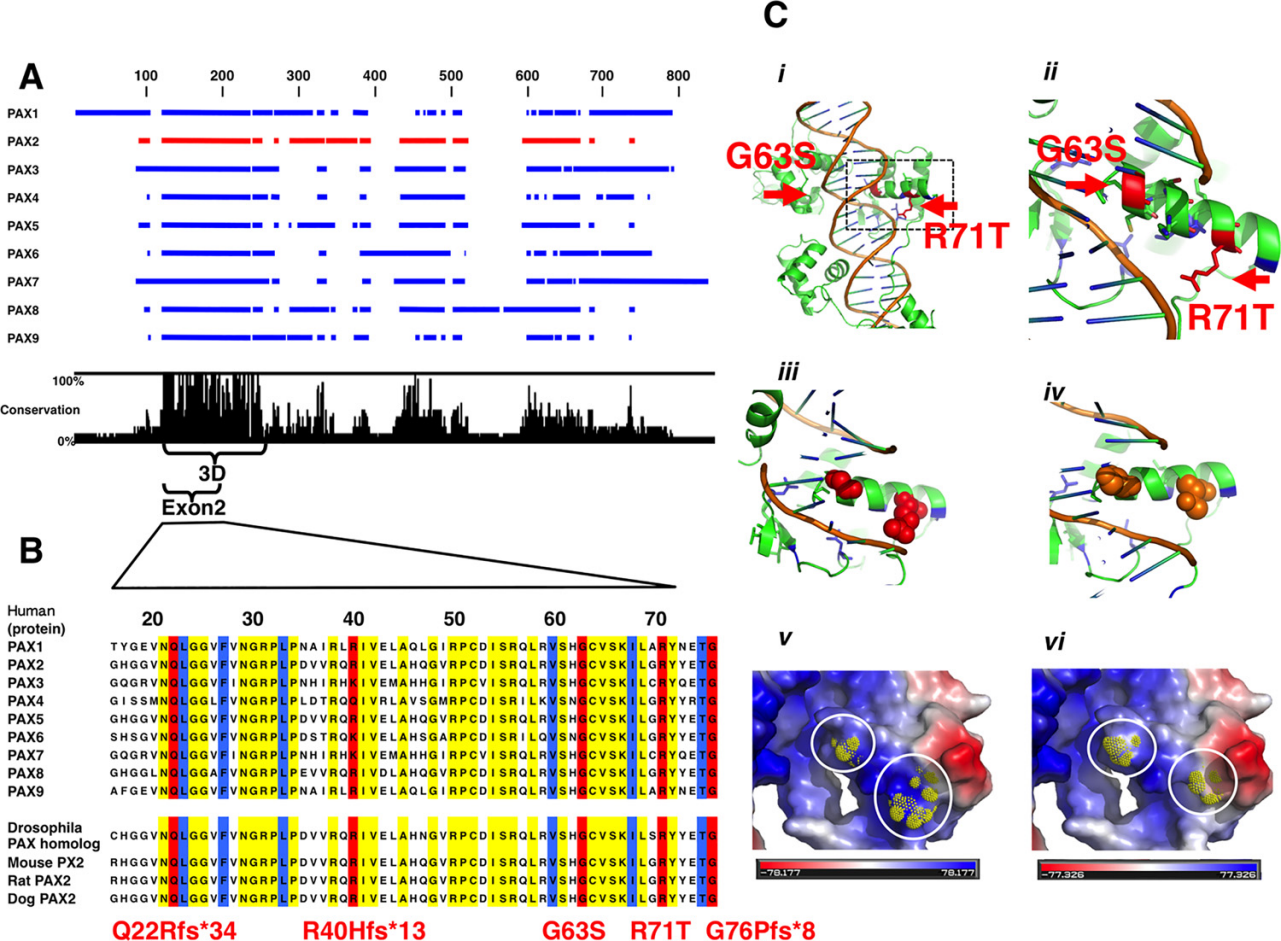
Mutations of *PAX2*. The paired domain of PAX family proteins (DNA binding domain) is well conserved among PAX family members (A). Panel B shows a partial amino acid sequence of the paired domain of PAX family proteins and *PAX2* from different species. Yellow bars indicate sequences with 100% identity with human *PAX1–9* and four different species. Red and blue bars indicate mutations identified in the present study and previous studies, respectively. Five mutation of *PAX2* were detected in exon 2. Three out of five mutations were insertion or deletion mutation in exon 2 with frameshifts. These frameshifts created new stop codons and thus generate nonsense mutations. Two other mutations were single nucleotide mutations (ex2 c.187G>A, G63S and ex2 c.212G>C, R71T). These two mutations are shown in panels C. The 3D-structure of the paired domain is shown in panel C*i*. The 3D-structure highlighted by the square in panel C*i* is magnified in panel C*ii*. Changes in structure and electric polarity by G63S and R71T mutations are shown in panels C*iii* to *vi*. Wild-type amino acids involved in DNA binding, glycine and arginine are shown in red. These two non-polar amino acid residues were mutated to the polar amino acid residues, serine and threonine, respectively. Blue and red highlighted amino acids in panels C*v* and *vi* are hydrophobic and hydrophilic, respectively.

Among candidate gene abnormalities, six *PAX2* mutations in 21 probands (28%; two in family cohorts [n = 5 and n = 2] and in 4 out of 19 patients with sporadic disease), including four novel mutations, were confirmed using Sanger sequencing (Table 1). Case 1–5 (a family) had the novel mutationc.119-120delGC (ClinVar accession number; SCV000255937). The novel heterozygous mutation ex2 c.187G>A (ClinVar accession number; SCV000255938) was detected in exon 2 of Patient 9, which generates the missense mutation G63S (Fig 1). Patients 10 and 11 had the novel *PAX2* mutations ex2, c.57-58insGTGAACC (ClinVar accession number; SCV000255939) and ex3, c.224-225insAC (ClinVar accession number; SCV000255940), respectively.

Mutations of *CHD7*, *SALL4*, *KIF26B*, and *SIX4* were confirmed using Sanger sequencing (S1 Fig). The *SALL4* variant (ex3 c.541G>A) was detected in Patient 8, and Patient 12 had multiple variants in *CHD7* (ex1 c.1565G>T), *KIF26B* (ex12 c.55146-5167dell ACCTCGCCCCCCAGCTCCGGGG) (ClinVar accession number; SCV000255941), and *SIX4* (ex1 c.1778A>T). Mutations were not detected in the other fourteen RCS cases without *PAX2* mutations and in four patients with only optic nerve coloboma.

### *PAX2* DNA recognition site and mutations in *PAX2* exon 2

The paired domain of PAX family proteins binds DNA and is well conserved among PAX proteins of humans (Fig 1A). The yellow bars in Fig 1B indicate 100% conservation of the amino acid sequences of *PAX1* to 9 of humans and *PAX2* of other species. Blue bars indicate known mutations, and the red bars indicate mutations in this study. Six mutation of PAX2 were detected in this study, and five of them were in exon 2. Three out of five mutations in exon 2 were insertion or deletion mutation with frameshifts. These frameshifts created new stop codons and thus generate nonsense mutations. Two other mutations were single nucleotide mutations (ex2 c.187G>A, G63S and ex2 c.212G>C, R71T) (Fig 1B). These mutations are located within the three-dimensional structure of the paired domain of PAX shown in Fig 1C. Blue and red indicate previously reported mutations and those discovered here, respectively. Red contact DNA and original amino acids are glycine and arginine (Fig 1C, S1 Movie and S2 Movie). These two non-polar amino acid residues (hydrophobic) were mutated to the polar (hydrophilic) amino acid residues serine and threonine, respectively. Mutation Taster was used to predict the pathogenicity of mutations. Almost all the identified mutations in our cases were found to have the potential to cause disease (Table 2).

**Table 2:** Gene mutation in renal coloboma syndrome.

			Data of Mutation Taster										Data of ExAC	
Case	Gene	Exon	Chromosome and position (hg 19)	DNA sequence change		AA changes	Score	Summary of Mutation Taster					dbSNP	Allele Frequency
**1**	**PAX2**	**2**	**chr10;102509578 102509579delGC**	**c.119-120delGC**	**cDNA. 669-670delGC**	R40Hfs*13	**N/A**	**Prediction disease causing**	**Amino acid sequence changed**	**Frameshift**	**Protein features (might be) affected**	**Splice site changes**	**Not reported**	**-**
**2**	**PAX2**	**2**	**chr10;102509578 102509579delGC**	**c.119-120delGC**	**cDNA. 669-670delGC**	R40Hfs*13	**N/A**	**Prediction disease causing**	**Amino acid sequence changed**	**Frameshift**	**Protein features (might be) affected**	**Splice site changes**	**Not reported**	**-**
**3**	**PAX2**	**2**	**chr10;102509578 102509579delGC**	**c.119-120delGC**	**cDNA. 669-670delGC**	R40Hfs*13	**N/A**	**Prediction disease causing**	**Amino acid sequence changed**	**Frameshift**	**Protein features (might be) affected**	**Splice site changes**	**Not reported**	**-**
**4**	**PAX2**	**2**	**chr10;102509578 102509579delGC**	**c.119-120delGC**	**cDNA. 669-670delGC**	R40Hfs*13	**N/A**	**Prediction disease causing**	**Amino acid sequence changed**	**Frameshift**	**Protein features (might be) affected**	**Splice site changes**	**Not reported**	**-**
**5**	**PAX2**	**2**	**chr10;102509578 102509579delGC**	**c.119-120delGC**	**cDNA. 669-670delGC**	R40Hfs*13	**N/A**	**Prediction disease causing**	**Amino acid sequence changed**	**Frameshift**	**Protein features (might be) affected**	**Splice site changes**	**Not reported**	**-**
**6**	**PAX2**	**2**	**chr10;102509671G>C**	**c.212G>C**	**cDNA. 762G>C**	**R71T**	**71**	**Prediction disease causing**	**Amino acid sequence changed**	**Known disease mutatio (pathogenic)**	**Protein features (might be) affected**	**Splice site changes**	**rs104894170**	**No data**
**7**	**PAX2**	**2**	**chr10;102509671G>C**	**c.212G>C**	**cDNA. 762G>C**	**R71T**	**71**	**Prediction disease causing**	**Amino acid sequence changed**	**Known disease mutatio (pathogenic)**	**Protein features (might be) affected**	**Splice site changes**	**rs104894170**	**No data**
**8**	**PAX2**	**9**	**chr10;102584439C>A**	**c.1023C>A**	**cDNA. 1573C>A**	Y341*	**6**	**Prediction disease causing**	**Amino acid sequence changed**		**Protein features (might be) affected**	**Splice site changes**	**rs78122364**	**3.30E-05**
8	**SALL4**	**3**	**chr20;50408481C>T**	**c.541G>A**	**cDNA. 653G>A**	**V181M**	**21**	**Prediction disease causing**	**Amino acid sequence changed**	**Heterozygous in TGP or ExAC**	**Protein features (might be) affected**	**Splice site changes**	**rs139382539**	**0.0004284**
**9**	**PAX2**	**2**	**chr10;102509646G>A**	**c.187G>A**	**cDNA. 737G>A**	**G63S**	**56**	**Prediction disease causing**	**Amino acid sequence changed**		**Protein features (might be) affected**	**Splice site changes**	**Not reported**	**-**
**10**	**PAX2**	**2**	**chr10;102509516 102509517insGTGAACC**	**c.57-58insGTGAACC**	**cDNA.607-608insGTGAACC**	Q22Rfs*34	**N/A**	**Prediction disease causing**	**Amino acid sequence changed**	**Frameshift**	**Protein features (might be) affected**		**Not reported**	**-**
**11**	**PAX2**	**3**	**chr10:102510462-102510463insAC**	**c.224-225insAC**	**cDNA.774-775insAC**	G76Pfs*8	**N/A**	**Prediction disease causing**	**Amino acid sequence changed**	**Frameshift**	**Protein features (might be) affected**	**Splice site changes**	**Not reported**	**-**
**12**	**KIF26B**	**12**	**chr1:245851431-245851452delACCTCGCCCCCCAGCTCCGGGG**	**c.5146-5167delACCTCGCCCCCCAGCTCCGGGG**	**cDNA.5586-5607delACCTCGCCCCCCAGCTCCGGGG**	T1716Pfs*13	**N/A**	**N/A**					**Not reported**	**-**
12	**CHD7**	**1**	**chr8;61655556G>T**	**c. 1565 G>T**	**cDNA. 2044G>T**	**G522V**	**109**	**Prediction disease causing**	**Amino acid sequence changed**	**Heterozygous in TGP or ExAC**	**Protein features (might be) affected**	**Splice site changes**	**rs142962579**	**0.002318**
12	**SIX4**	**1**	**chr14;61180693T>A**	**c.1778A>T**	**cDNA. 1828A>T**	**N593I**	**149**	**Prediction polymorphism**	**Amino acid sequence changed**	**Heterozygous in TGP or ExAC**	**Protein features (might be) affected**	**Splice site changes**	**rs77115877**	**0.002656**

Mutation Taster (http://www.mutationtaster.org) was used to predict potential contributions of individual mutations to disease. Allele frequencies were evaluated using the ExAc database (http://exac.broadinstitute.org). N/A; not appropriate. The score of Mutation Taster is taken from the Grantham Matrix for amino acid substitutions and reflects the physicochemical difference between the original and the mutated amino acid. It ranges from 0.0 to 215 but does not provide a value for amino acid insertions/deletions. A higher Grantham score is indicative of a greater difference in chemical properties between two amino acids and can indicate a stronger effect on protein structure and function.

### Clinical manifestation of RCS with or without *PAX2* mutations

**Fig 2:**
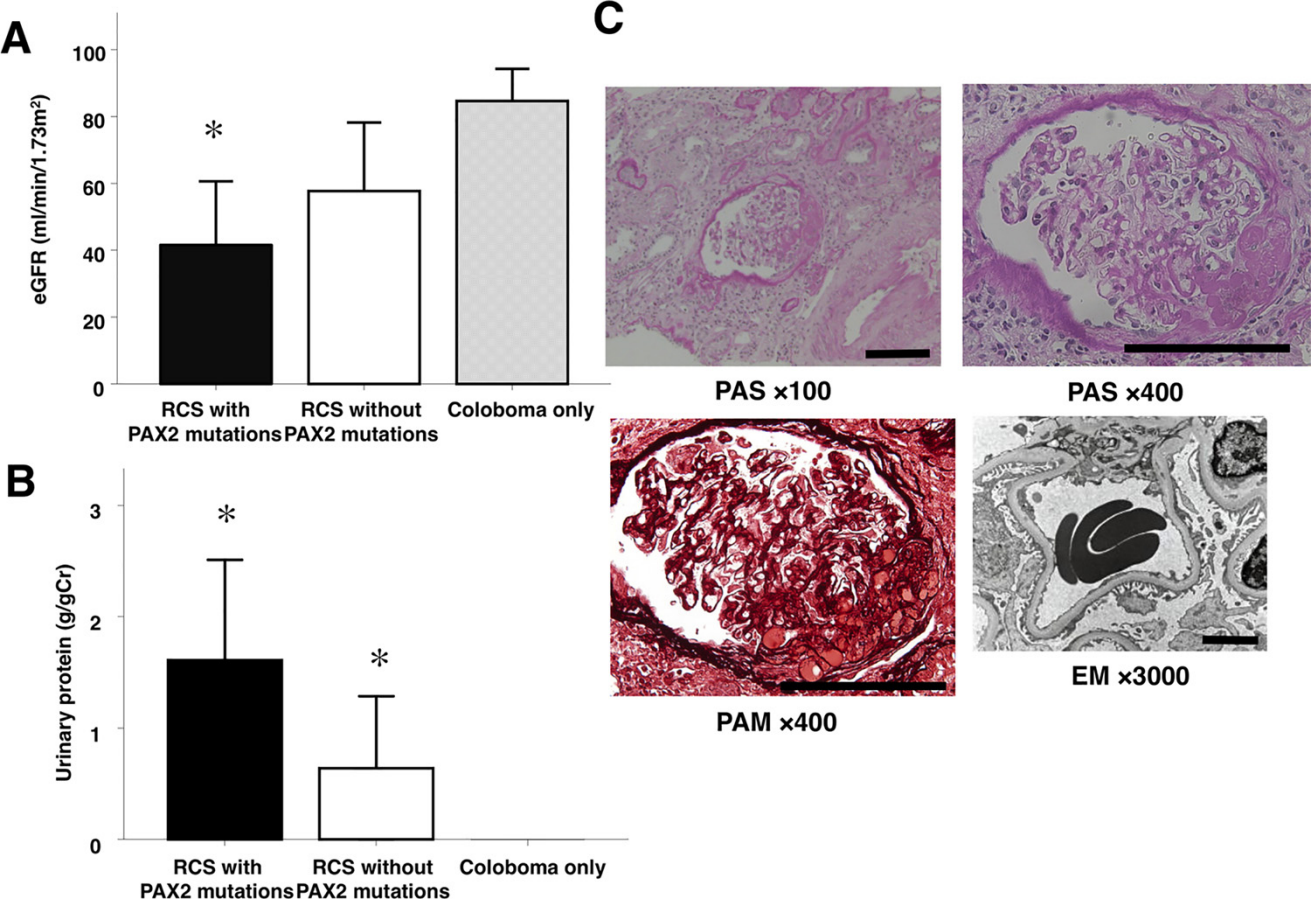
Clinical manifestation of kidney in patients with *PAX2* mutations. *PAX2* mutations substantially contribute to the renal manifestations of RCS. In patients without dialysis or kidney transplantation, estimated GFR were lower in patients with *PAX2* mutations (A), and proteinuria was more severe in patients with *PAX2* mutations (B). Histology of a renal biopsy specimen from Patient 8 is shown in panel C.A representative segmental sclerosing lesion is observed at a glomerulus by PAS (*i*, *ii*) and PAM (*iii*) staining. Original magnifications; panel *I*, ×100; planes *ii* and *iii*, ×400. Panel *iv* shows a representative image from transmission electron microscopy at an original magnification of ×3,000. Foot process effacement is observed without evidence of immune deposits. Scale bars represent 100 μm in light microscopy images and 5 μm in electron microscopy images. Values represent the mean ± SEM. *, p < 0.05 vs. “Coloboma only”. “RCS with PAX2 mutations” indicates clinically diagnosed renal-coloboma syndrome cases with *PAX2* gene mutations (n = 5), “RCS without PAX2 mutations” indicates clinically diagnosed renal-coloboma syndrome cases without *PAX2* gene mutations (n = 13), and “Coloboma only” indicates cases with optic nerve coloboma and no kidney abnormality (a disease control; n = 4).

Among twenty-six patients clinically diagnosed with RCS, six probands (28%) had *PAX2* mutations (Table 2). *PAX2* mutations were undetectable in the other fifteen patients (57.7%) who were clinically diagnosed with RCS. Although 54.5% of patients with *PAX2* mutations received hemodialysis, only 13.3% of those without *PAX2* mutations received dialysis or received a kidney transplant (Table 3). Estimated GFR was lower in patients with *PAX2* mutations (Fig 2A). Proteinuria was more severe in patients with *PAX2* mutations (Fig 2B).

**Table 3:** Clinical manifestation of renal coloboma syndrome with/without PAX2 mutation.

	Case	Age	Gender	Coloboma			Kidney		
				Rt	Lt	total	Gross morphology	Pathology	Function
**With** ***PAX2* mutation**	**1**	**44**	**F**	**1**	**3**	**4**	**Normal**	**ND**	**HD**
**With** ***PAX2* mutation**	**2**	**18**	**M**	**1**	**1**	**2**	**Malrotation**	**Glomerulomegaly**	**Cr 1.23**
**With** ***PAX2* mutation**	**3**	**61**	**F**	**1**	**1**	**2**	**Hypoplasia/atrophy**	**ND**	**HD**
**With** ***PAX2* mutation**	**4**	**59**	**F**	**3**	**4**	**7**	**Malrotation**	**ND**	**Cr 1.08**
**With** ***PAX2* mutation**	**5**	**61**	**F**	**1**	**2**	**3**	**Hypoplasia/atrophy**	**ND**	**HD**
**With** ***PAX2* mutation**	**6**	**34**	**F**	**3**	**3**	**6**	**Malrotation**	**TIN**	**HD**
**With** ***PAX2* mutation**	**7**	**61**	**F**	**3**	**2**	**5**	**Hypoplasia/atrophy**	**ND**	**HD**
**With** ***PAX2* mutation**	**8**	**28**	**F**	**1**	**3**	**4**	**ML, lt. double ureters**	**FSGS**	**Cr 1.47**
**With** ***PAX2* mutation**	**9**	**37**	**F**	**3**	**1**	**4**	**Hypoplasia/atrophy**	**Mes PGN**	**Cr 2.20**
**With** ***PAX2* mutation**	**10**	**14**	**M**	**3**	**3**	**6**	**Normal**	**FSGS**	**Cr 1.41**
**With** ***PAX2* mutation**	**11**	**38**	**M**	**4**	**4**	**8**	**Hypoplasia/atrophy**	**FSGS**	**HD**
**Without** ***PAX2* mutation**	**12**	**31**	**F**	**1**	**1**	**2**	**Rt. hypoplasia/atrophy**	**ND**	**Transplantation**
**Without** ***PAX2* mutation**	**13**	**65**	**M**	**0**	**1**	**1**	**Malrotation**	**IgA nephropaty**	**Cr 1.27**
**Without** ***PAX2* mutation**	**14**	**76**	**F**	**3**	**3**	**6**	**Malrotation**	**Glomerulomegaly**	**Cr 0.50**
**Without** ***PAX2* mutation**	**15**	**27**	**M**	**1**	**1**	**2**	**Malrotation**	**Glomerulomegaly**	**Cr 1.80**
**Without** ***PAX2* mutation**	**16**	**11**	**F**	**1**	**1**	**2**	**Malrotation**	**ND**	**Cr 0.50**
**Without** ***PAX2* mutation**	**17**	**44**	**F**	**1**	**0**	**1**	**Malrotation**	**ND**	**Cr 0.60**
**Without** ***PAX2* mutation**	**18**	**51**	**F**	**0**	**1**	**1**	**Hypoplasia/atrophy**	**ND**	**Cr 1.07**
**Without** ***PAX2* mutation**	**19**	**65**	**M**	**1**	**0**	**1**	**Hypoplasia/atrophy**	**ND**	**Cr 1.97**
**Without** ***PAX2* mutation**	**20**	**43**	**F**	**1**	**0**	**1**	**Hypoplasia/atrophy**	**ND**	**Cr 4.31**
**Without** ***PAX2* mutation**	**21**	**37**	**M**	**1**	**0**	**1**	**Hypoplasia/atrophy**	**MCNS**	**Cr 2.26**
**Without** ***PAX2* mutation**	**22**	**60**	**F**	**0**	**1**	**1**	**Malrotation**	**ND**	**Cr 0.69**
**Without** ***PAX2* mutation**	**23**	**66**	**M**	**1**	**0**	**1**	**Rt. hypoplasia/atrophy**	**ND**	**Cr 1.57**
**Without** ***PAX2* mutation**	**24**	**52**	**F**	**1**	**0**	**1**	**Rt. hypoplasia/atrophy**	**ND**	**CAPD**
**Without** ***PAX2* mutation**	**25**	**47**	**M**	**1**	**1**	**2**	**Malrotation**	**ND**	**Cr 1.17**
**Without** ***PAX2* mutation**	**26**	**63**	**M**	**1**	**1**	**2**	**Rt. hypoplasia/atrophy**	**ND**	**Cr 0.83**
**Coloboma only**	**27**	**29**	**F**	**1**	**1**	**2**	**Normal**	**ND**	**Cr 0.70**
**Coloboma only**	**28**	**30**	**F**	**1**	**0**	**1**	**Normal**	**ND**	**Cr 0.60**
**Coloboma only**	**29**	**73**	**F**	**1**	**1**	**2**	**Normal**	**ND**	**Cr 0.60**
**Coloboma only**	**30**	**65**	**F**	**1**	**0**	**1**	**Normal**	**ND**	**Cr 0.51**

Optic nerve coloboma of each eye was scored 0 to 4 (Score 0, normal optic disc to score 4, severe c olobomma). Kidney abnormality of gross morphology, pathology, and function are summarized. ND; not done, FSGS; focal segmental glomerulosclerosis, Mes PGN; mesangial proliferative glomerulonephritis, MCNS; minimal change nephrotic syndrome.

Kidney biopsies were acquired from nine of twenty-six clinically diagnosed patients with RCS. Five patients had *PAX2* mutations, and *PAX2* mutations were undetectable in four patients. Three of five patients with *PAX2* mutations had FSGS (Table 3, Fig 2C), and the other two had glomerulomegaly or non-IgA mesangial proliferative glomerulonephritis. However, the four patients without *PAX2* mutations did not have FSGS, two had glomerulomegaly, and one each had IgA nephropathy or minimal change nephrotic syndrome.

**Fig 3:**
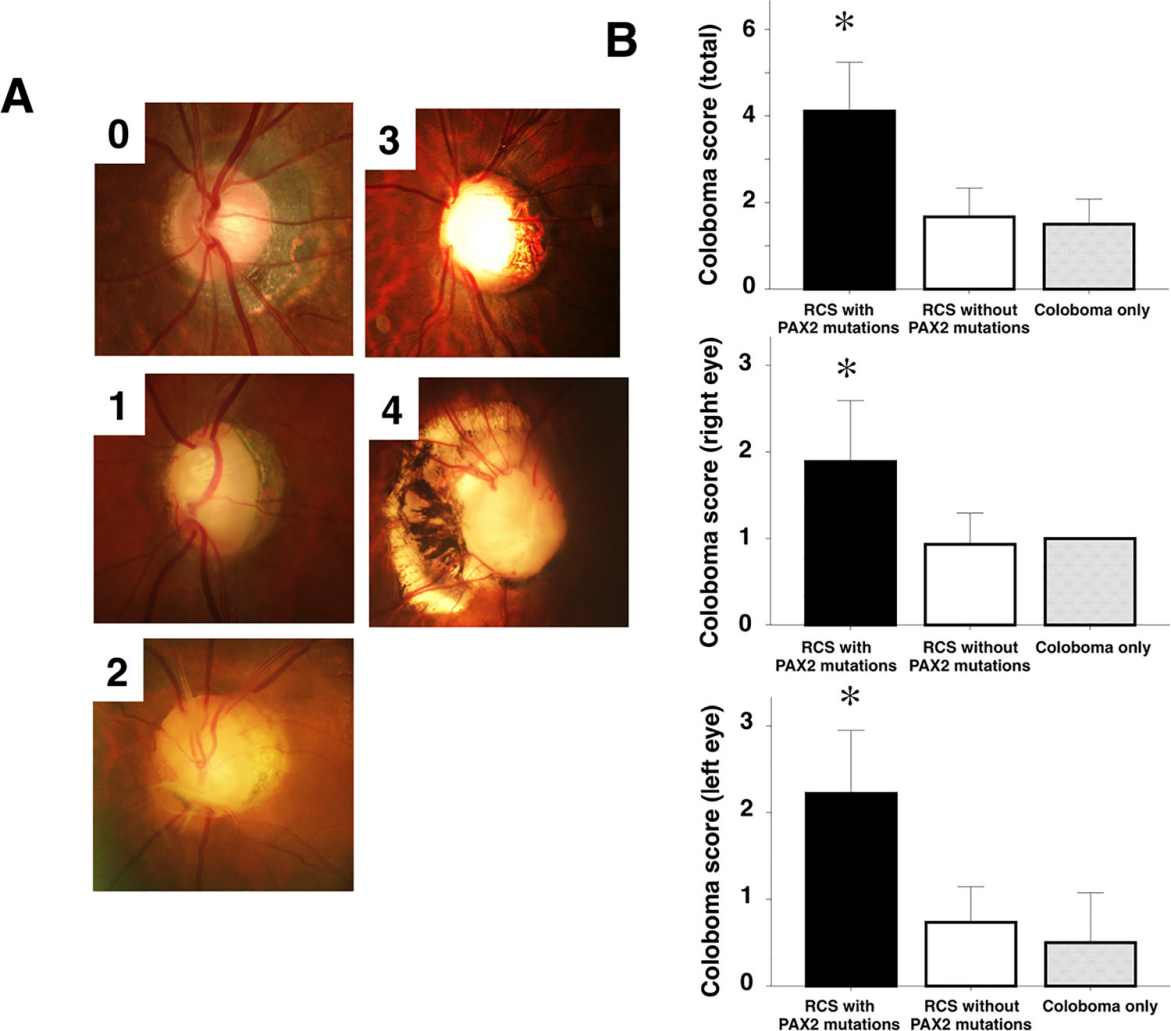
Optic nerve coloboma was severe in patients with *PAX2* mutations. Representative images of coloboma scores 0–4 are shown in *panel A*. Score 0, normal optic disc; Score 1, optic disc dysplasia with an unusual pattern of the retinal vessels and cilioretinal arteries; Score 2, optic disc pit associated with vascular abnormalities and a cilioretinal artery; Score 3, large coloboma involving the entire surface of the optic disc; Score 4, large coloboma of the optic disc and adjacent retina, or morning glory anomaly (with radial emergence of the retinal vessels). The mean coloboma scores of each group are shown in *panel B*. The coloboma scores (total, right and left eye) were significantly higher in patients with *PAX2* mutations. Values represent the mean ± SEM. *, p < 0.05 vs. coloboma scores of patients without *PAX2* mutations. “RCS with PAX2 mutations” indicates clinically diagnosed renal-coloboma syndrome cases with *PAX2* gene mutations (n = 11), “RCS without PAX2 mutations” indicates clinically diagnosed renal-coloboma syndrome cases without *PAX2* gene mutations (n = 15), and “Coloboma only” indicates cases with optic nerve coloboma and no kidney abnormality (a disease control; n = 4).

Thirteen and six patients had bilateral or monolateral typical ocular coloboma, respectively. The coloboma scores were significantly higher in patients with *PAX2* mutations (Table 3, Fig 3). However, the degree of kidney dysfunction and coloboma varied, even among patients with the same *PAX2* mutation.

Patients 8 and 12 had variants in other genes. Patient 8 had *SALL4* variant. Patient 12 with severe kidney dysfunction had three variants (*KIF26B*, *CHD7*, and *SIX4*) and subsequently received a kidney transplant (Tables 2 and 3).

### Familial cases with *PAX2* mutations

**Fig 4:**
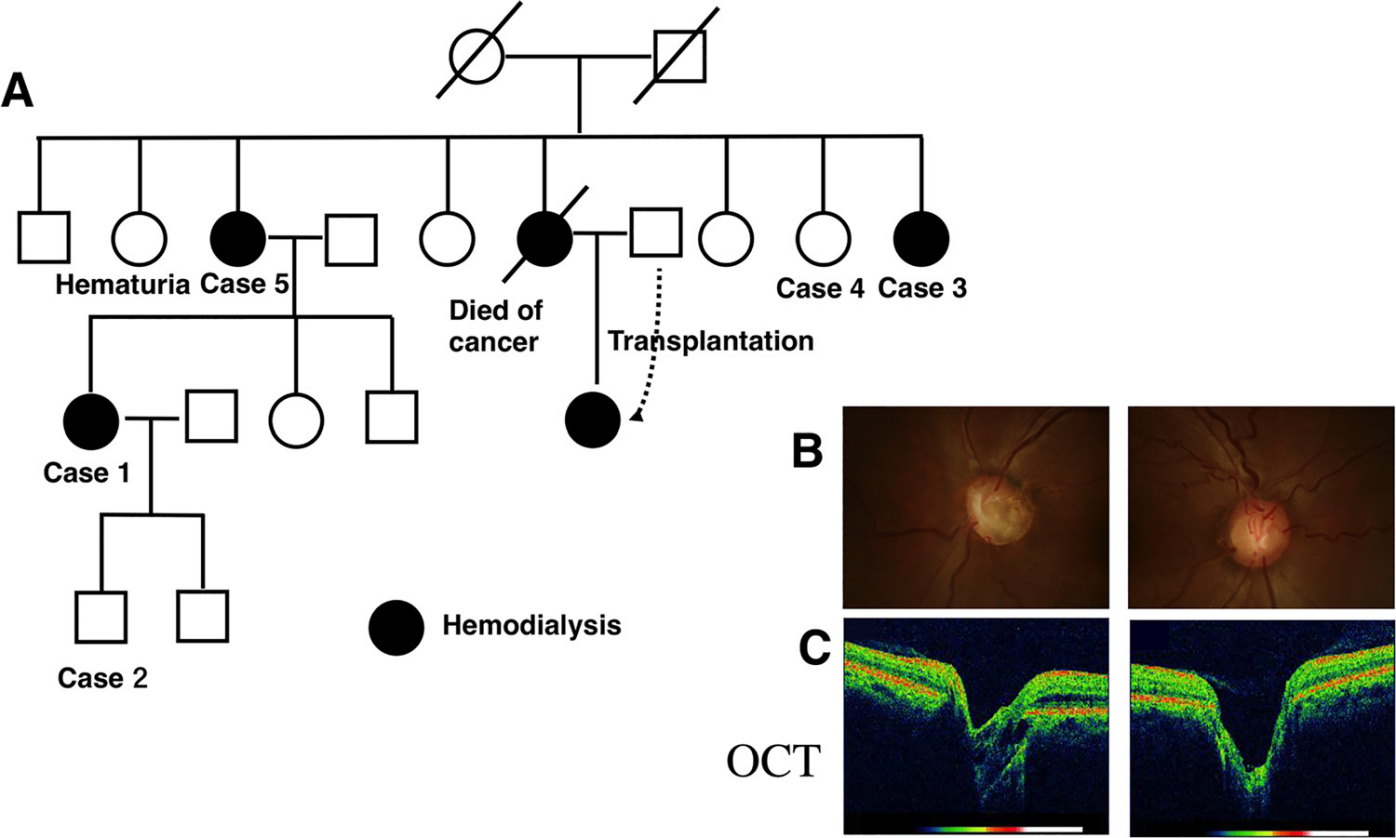
Pedigree of the family with RCS with *PAX2* mutations. Pedigree of the family with RCS showing classical autosomal dominant inheritance across three generations. ☐, males; ❍, females; ●, female patients receiving hemodialysis. A diagonal line through a symbol means a patient is deceased.Ocular fundal images of Patient 1 (B, C) indicate that she had ocular coloboma (coloboma scores: right eye = 1, left eye = 3). Cross-sectional images of the optic disc acquired using optical coherence tomography (OCT) show deep excavation in both eyes (C).

Patients 1–5 represented one family cohort with the same novel *PAX2* mutation ex2 c. 119-120delGC (Table 2, Fig 4). Patients 1, 3, and 5 received hemodialysis. Although Patient 4 was the sister of Patients 3 and 5, who all had the same *PAX2* mutation, her kidney dysfunction was mild (Cr 1.24 mg/dl). Patient 1 had a visual disability with some visual field defects. She and her son (Patient 2, 18 years of age) had bilateral optic nerve coloboma, and they each had the same *PAX2* mutation. The left eye of Patient 1 was the most severely affected (coloboma scores: Patient 1, right eye score = 1, left eye score = 3; Patient 2, right eye score = 1, left eye score = 2 (Fig 4).

The second family cohort included Patient 6 and her mother, Patient 7. They had a heterozygous *PAX2* mutation c.212G>C in exon 2 that creates the missense mutation R71T. Patient 6 previously reported as a case of acro-renal-ocular syndrome [17]. These patients received hemodialysis and had very severe ocular coloboma (coloboma scores: Patient 6, right eye score = 3, left eye score = 3; Patient 7, right eye score = 3, left eye score = 2).

## Discussion

In the present study, we screened the nucleotide sequences of *PAX2* and those of 25 other genes and identified *PAX2* mutations, including four mutations that are novel, in 6 out of 21 probands who were clinically diagnosed with RCS (28%). Moreover, we detected four sequence variants in other genes, including a novel KIF26B mutation.*PAX2* mutations manifest as clinical abnormalities of the kidneys and eyes, and mutations in other genes may exert the same effects. The most significant findings of the present study are as follows: 1. Mutations in genes other than *PAX2* may contribute to the pathogenesis of RCS; 2. We present an analysis of a series of kidney biopsies of patients with RCS; and 3. Factors other than *PAX2* mutations may contribute to the progression of kidney and eye abnormalities in patients with RCS.

Of the 21 probands with RCS characterized here, 6 (28%) had PAX2 mutations, and two further patients had sequence variants in other genes, including one patient with a novel KIF26B mutation. Approximately 50% cases of RCS have no *PAX2* mutations[14]; however, no other mutations are reported. We detected four non-PAX2 sequence variants in our cases of RCS. Among them, we detected *KIF26B* mutation that have not been reported, to our knowledge, in patients with RCS. The allele frequency of CHD7, SIX4, and SALL4 sequence variants were 0.04–0.2% and may therefore represent polymorphisms. Therefore, these sequence variants would not be considered to be pathogenic[18, 19]. In contrast, *KIF26B*, which encodes a member of the kinesin family of motor proteins, exerts a major effect on development [20]. Thus, *KIF26B*-deficient mice exhibit neonatal lethality with impaired kidney development towing to the loss of cortical nephrogenic zone mesenchyme and the failure of ureteric buds to invade and branch into the mesenchyme. To the best of our knowledge, the present study is the first to detect a *KIF26B* mutation in humans with kidney dysfunction. The novel *KIF26B* sequence variant, which was a 22bp deletion affecting exon 12 of *KIF26B*, and was predicted to cause a frameshift mutation prior to the termination of the coding region of *KIF26B* in exon 15, would be damaging to the protein. Because patient 12 with the KIF26B variant did not have a PAX2 mutation, this mutation is potentially the causative mutation in this patient. Our patient with the *KIF26B* mutation developed kidney dysfunction and eventually received a kidney transplant. Further studies of renal coloboma syndrome that analyze exosome or whole genome sequences are required to detect gene mutation in this syndrome and mutation in other genes of patients with RCS.

Our analysis of kidney biopsies from nine of 26 patients with RCS reveal that five harbor*PAX2* mutations. Three of five patients with *PAX2* mutations, but not those without, exhibited FSGS. Previous analyses of the biopsies of patients indicate that re-expression of *PAX2* by podocytes is an early marker of FSGS [21]. Moreover, proliferating parietal epithelial cells that express *PAX2* and cytokeratin may contribute to FSGS [22]. Moreover, a *PAX2* mutation contributes to adult-onset FSGS, and 4% of patients with FSGS harbor the *PAX2* mutation [23]. Here we show that 60% of patients with *PAX2* mutations had FSGS, indicating that although the mechanisms of FSGS progression are not defined, *PAX2* may play a critical role in disease progression.

We report here two families with PAX2 mutations (Case1 to 5 and Case6, 7) in this manuscript. Each member of one family (Case1 to 5) had one PAX2 mutation (c.119-120delGC), and each member of another family (Case6 and 7) had the other PAX2 mutation (c.212G>C). Although each family member had the same *PAX2* mutation, the extent of kidney dysfunction differed significantly among them. Vesicoureteral reflux (VUR) is an important factor when considering determinants of renal dysfunction of patients with RCS [24]. *PAX2* mutations contribute to the kidney and urinary tract development, and participate in VUR. Minor VUR can cause renal failure and kidney scarring over time. In addition to various pathological glomerular changes, the severity of VUR may explain the differences in kidney dysfunction between subjects with the same *PAX2* mutation.

Ocular involvement is present in patients with *PAX2* mutations [25, 26], and bilateral optic nerve coloboma is typically associated with RCS. Optic nerve or disc dysplasia, microphthalmia, morning glory anomaly, optic nerve cysts, scleral staphyloma, myopia, nystagmus, and cataracts were observed as well [27, 28]. Our data reveal that optic nerve coloboma was more severe in patients with *PAX2* mutations. However, the degree of optic nerve coloboma varied between patients or between each eye of an individual, although patients had the same *PAX2* mutation. Moreover, a recent study reveals that even identical twins with *PAX2* mutations show different eye and kidney abnormalities [29]. Consistent with the present data, others suggest that point mutations within the coding region of *PAX2* may not represent the only cause of RCS [14].These findings indicate that other factors may exert additive effects on the progression of kidney and eye dysfunctions.

This study had some limitations. Parental studies are necessary to understand the contribution of genetic mutations to clinical phenotypes. However, the approved protocol of the present study did not include parents without the clinical manifestations of RCS. Therefore, we aim to include parental studies in our future studies. Secondly, family bias may have been present in this study. As RCS is very rare, we included as many cases as possible. Therefore, two families (of 5 and 2 cases, respectively) were included in this study. As there were significant differences in clinical ocular and renal manifestations between individual family members with identical gene mutations [23, 30, 31], family bias may have affected the analysis conducted in the present study. Moreover, the patient numbers in this study are very small, and that even just a small phenotypic bias can sway the analysis one way or the other. Therefore conclusions from this study can only be very tentative. Additional familial cases are required to adequately reduce this potential source of bias.

Our data indicate that *PAX2* mutation is a key abnormality in RCS and may make a major contribution to the pathogenesis of kidney and eye abnormalities. However, other factors and gene mutations may play a role, and further human and animal studies will be required to define the mechanism of pathogenesis of RCS.

## Supporting Information

S1 Fig Mutations of *KIF26B*, *CHD7*, *SIX4*, *and SALL4*. To detect mutations associated with renal coloboma syndrome (RCS), 26 patients with RCS and 4 patients with optic nerve coloboma only were screened using next-generation sequence analysis. Detected candidate mutations were confirmed using Sanger sequencing. Mutation **c.5146-5167delACCTCGCCCCCCAGCTCCGGGG** in *KIF26B* was detected in exon 12. Similarly, mutation c.1565 G>T in *CHD7* was detected in exon 1, mutation c.569 T>A in *SIX4* was detected in exon 1, and mutation c.2814 C>T in *SALL4* was detected in exon 3.
(PPT)

S1 Movie 3D-structure movie of the *PAX2* paired domain binding to DNA. 3D-structure images are shown in Fig 1 C*i-iv* and C*v-vi*, respectively. The 3D-structure of the paired domain is shown in S1 Movie. Changes in structure by G63S and R71T mutations are shown. Wild-type amino acids involved in DNA binding, glycine and arginine, are shown in red. These two non-polar amino acid residues were mutated to the polar amino acid residues, serine and threonine, respectively.
(MOV)

S2 Movie 3D-electric polarity movie of the *PAX2* paired domain binding to DNA. 3D-structure images are shown in Fig 1 C*i-iv* and C*v-vi*, respectively. The 3D-structure of the paired domain is shown in S2 Movie. Changes in electric polarity by G63S and R71T mutations are shown. Amino acids highlighted in blue and red are hydrophobic and hydrophilic, respectively.
(MOV)
