# Supplementary material for: Association of PAX2 and Other Gene Mutations with the Clinical Manifestations of Renal Coloboma Syndrome
Source: PLoS One. 2015 Nov 16;10(11):e0142843. doi: 10.1371/journal.pone.0142843 (PMC4646464; doi:10.1371/journal.pone.0142843)
Supplement: S1 Fig — To detect mutations associated with renal coloboma syndrome (RCS), 26 patients with RCS and 4 patients with optic nerve coloboma only were screened using next-generation sequence analysis. Detected candidate mutations were confirmed using Sanger sequencing. Mutation c.5146-5167delACCTCGCCCCCCAGCTCCGGGG in KIF26B was detected in exon 12. Similarly, mutation c.1565 G>T in CHD7 was detected in exon 1, mutation c.569 T>A in SIX4 was detected in exon 1, and mutation c.2814 C>T in SALL4 was detected in exon 3. (PPT) [file pone.0142843.s001.ppt]

## Slide 1
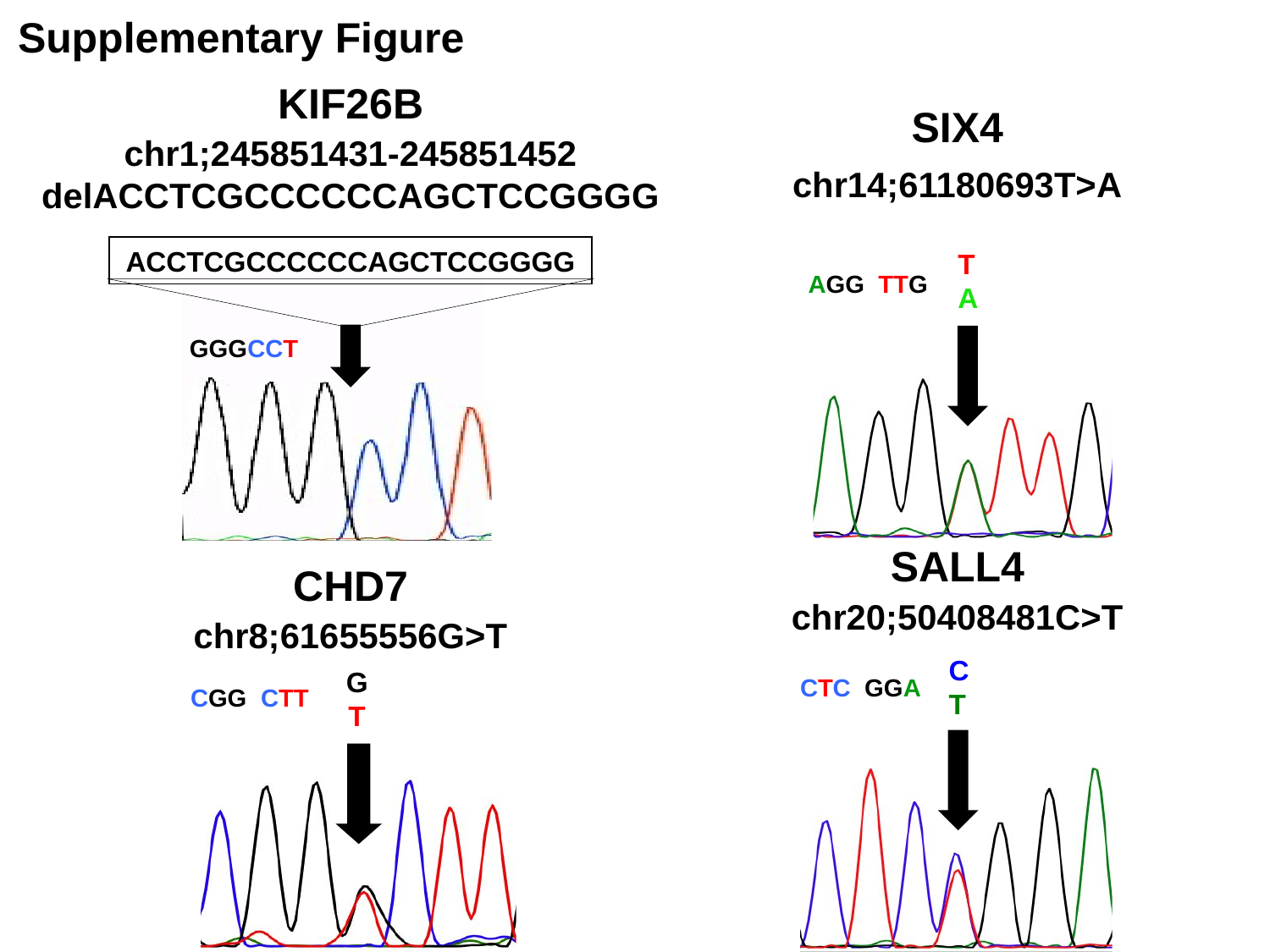

Supplementary Figure
KIF26B
SIX4
chr1;245851431-245851452
delACCTCGCCCCCCAGCTCCGGGG
chr14;61180693T>A
ACCTCGCCCCCCAGCTCCGGGG
T
A
AGG TTG
GGGCCT
SALL4
CHD7
chr20;50408481C>T
chr8;61655556G>T
CT
CTC GGA
G
T
CGG CTT
